# The assembly effect: the connectedness between populations is a double‐edged sword for public health interventions

**DOI:** 10.1186/s12936-021-03726-x

**Published:** 2021-04-17

**Authors:** Sai Thein Than Tun, Daniel M. Parker, Ricardo Aguas, Lisa J. White

**Affiliations:** 1 Mahidol-Oxford Tropical Medicine Research Unit, Faculty of Tropical Medicine, Mahidol University, Nakhon Ratchasima, Thailand; 2 Centre for Tropical Medicine and Global Health, Nuffield Department of Medicine, University of Oxford, Oxford, UK; 3 Department of Population Health and Disease Prevention, University of California, Irvine, USA; 4 Department of Epidemiology, University of California, Irvine, USA; 5 Li Ka Shing Centre for Health Information and Discovery, Nuffield Department of Medicine, Big Data Institute, University of Oxford, Oxford, UK

**Keywords:** Herd immunity, Malaria, Public health, Immunizing infections, Mathematical modelling

## Abstract

**Background:**

Many public health interventions lead to disruption or decrease of transmission, providing a beneficial effect for people in the population regardless of whether or not they individually participate in the intervention. This protective benefit has been referred to as a herd or community effect and is dependent on sufficient population participation. In practice, public health interventions are implemented at different spatial scales (i.e., at the village, district, or provincial level). Populations, however defined (i.e., neighbourhoods, villages, districts) are frequently connected to other populations through human movement or travel, and this connectedness can influence potential herd effects.

**Methods:**

The impact of a public health intervention (mass drug administration for malaria) was modelled, for different levels of connectedness between populations that have similar disease epidemiology (e.g., two nearby villages which have similar baseline malaria incidences and similar malaria intervention measures), or between populations of varying disease epidemiology (e.g., two nearby villages which have different baseline malaria incidences and/or malaria intervention measures).

**Results:**

The overall impact of the interventions deployed could be influenced either positively (adding value to the intervention) or negatively (reducing the impact of the intervention) by how much the intervention units are connected with each other (e.g., how frequent people go to the other village or town) and how different the disease intensity between them are. This phenomenon is termed the “assembly effect”, and it is a meta-population version of the more commonly understood “herd effect”.

**Conclusions:**

The connectedness of intervention units or populations is an important factor to be considered to achieve success in public health interventions that could provide herd effects. Appreciating the assembly effect can improve the cost-effective strategies for global disease elimination projects.

**Supplementary Information:**

The online version contains supplementary material available at 10.1186/s12936-021-03726-x.

## Background

Communicable diseases made up 44 and 31% of mortality in low and low-middle income countries as of 2017 [1]. Public health interventions have been used for the control and prevention of diseases. Whenever a large enough proportion of the population take up an effective public health intervention for a communicable disease, the transmission of that disease will be reduced and there can be a community-level effect commonly referred to as the “herd effect” [2]. This herd effect provides a protective benefit to all members of a population, regardless of individual participation in the intervention. Conversely, when relatively few individuals in a population participate in an intervention there will be a negligible impact on transmission and, therefore, no herd effect.

Herd effects have been documented for several interventions that reduce the transmission potential such as early detection and treatment of pulmonary tuberculosis, mass drug administration (MDA) against lymphatic filariasis [2], insecticide-treated nets (ITN) against malaria infections [3] and recently for MDA against *Plasmodium falciparum* malaria [4]. Herd effects depend on sufficient population adherence to an intervention in order to provide a protective benefit to all individuals in the population. This threshold of participation has been considered in the context of a single population, with little consideration of the existence of meta-populations (groups of spatially separated populations of the same species which interact at some level [5]). Here, how connectedness with other populations from different areas influences the effectiveness of the public health interventions was explored, by using malaria elimination as a working example.

Types of intervention for effective malaria control depend on the level of malaria transmission [6–8]. In high burden malaria areas, malaria control measures, such as indoor residual spraying (IRS), insecticide-treated mosquito nets (ITNs/LLINs), and ensuring universal access to malaria prevention, diagnosis and treatment aim to reduce malaria prevalence. Population-wide parasite clearance by mass drug administration (MDA) could be used to accelerate the malaria elimination process. Investigation and treatment of residual cases should be done only when the malaria transmission intensity is low enough. Progression from malaria control to malaria elimination is a continuous process with different countries, subnational areas, and communities at different stages on the pathway towards malaria elimination [8]. To address the uneven landscape of malaria transmission in different areas, risk maps can be created through the combination of epidemiological data, geographical information system, and remote sensing of environmental features, followed by a stratification algorithm to allow for better targeting and improved efficiency of malaria interventions [8–10]. Targeting high-risk areas would definitely have a high impact, but when the goal is the global elimination of malaria, the connectedness of the geographical areas through human and/or mosquito movement must also be taken into account. For example, a population movement survey done in the Thai-Myanmar border area found that 44% of participants in one malaria cluster crossed the international border at least once a month [11]. The two countries have different healthcare infrastructures and malaria transmission intensities [11, 12] and such connectedness could negatively impact the malaria elimination efforts on one side provided that no similar malaria elimination effort (e.g. mass drug administration, increased access to early diagnosis and treatment) is made across the border. Previous models have also suggested the importance of taking into consideration human movement for efficient deployment of malaria interventions [13, 14].

A theoretical framework with two interconnected populations, hereafter referred to as “patches”, is presented here. Connectedness in the model is the abstraction of human mobility between patches causing humans to contribute to the infectious/ non-infectious pool of individuals in his/her non-native patches. As an example, when a person from patch 1 spends some proportion of one’s time in patch 2, that person will partially contribute towards the force of infection of a disease in patch 2, either augmenting or diluting it, depending on one’s disease transmissibility status. How the magnitude of the connectedness between two patches impacts the potential success of MDA deployment in each of them is explored. First, the model was validated against the empirical results from a detailed MDA pilot study [4]. And it was used to predict the outcomes of a series of alternative scenarios for different connectedness, different transmission levels, and different intervention coverages to obtain a more complete picture of this phenomenon and its implications.

The goal of this study was to evaluate the impact of human movement or travel on the success of public health interventions that could produce herd effects. In particular, anti-malarial MDA, under a range of different malaria burden and human movement scenarios was explored. This work has operational relevance for targeted anti-malarial campaigns, especially with regard to the spatial unit (household, village, district) that is being targeted. It also has relevance for other public health interventions, all of which have an inherent spatial unit that is being targeted.

## Methods

All simulations and analyses were carried out using the R software version 3.6.0 [15] with the following packages: deSolve [16], Rcpp [17], and lattice [18]. A two-patch model was developed as an extension of a previously published single-patch model [19]. Each patch had 8 compartments, representing the subgroups with different characteristics such as susceptibility and infectiousness of malaria. There were two types of susceptible compartments: Susceptible with active antimalarial drug (S_D_), and those without drug (S). Likewise, there were two types of recovered compartments: R_D_ and R. Individuals in the compartments with active drugs were immune to infection until the drugs run out from the body. The infectious compartment was separated into three sub-compartments: I_C_ represented clinical cases, I_A_ represented the cases that were asymptomatic, but detectable through microscopy and rapid-diagnostic test (RDT), and I_U_ represented the cases that were asymptomatic and undetectable through microscopy and RDT. For brevity, all sub-compartments of I were combined as an I in the subsequent equation and figure. There was a treatment compartment (T) to accommodate those from the infectious compartment who got treated. The natural progression of malaria in the model was from S to I to R to S. When MDA was implemented, a proportion of the population under coverage received protection from the disease for some duration (i.e., Individuals from S & R were moved to their respective compartments with active drug, S_D_ & R_D_, where they would remain until the prophylactic effect of the drug was lost. Individuals from I were moved to T).

**Fig. 1:**
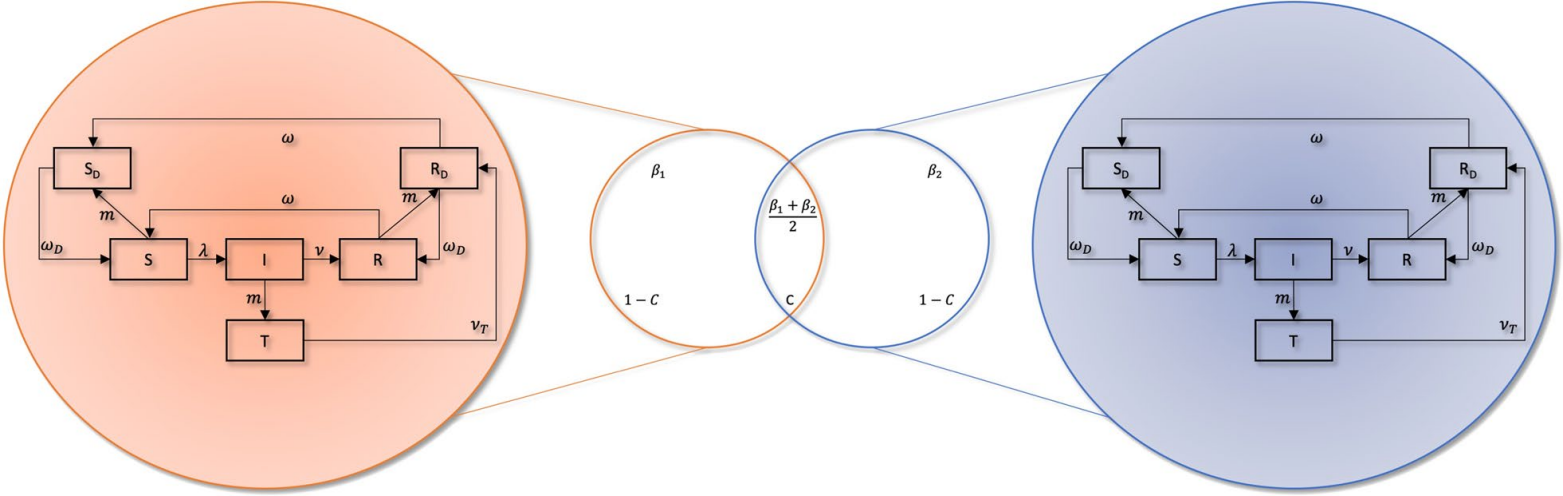
Two-patch compartmental model. $C$ indicates the proportion of the population in each patch that shares a common infectious reservoir with the other patch. When the two patches are isolated (i.e., not at all connected, C = 0), they share no infections and each individual’s risk of infection in a patch is completely independent of that in the other patch. At the other extreme of the connectedness spectrum (C = 1), all individuals in the two patches are subject to the same force of infection (λ). β is the effective biting rate adjusted by vector interventions. Zoomed-in areas describe the simplified compartments within each patch- S: Susceptible. $\,\,\,\,\,I:\,\,\,\,$ Infected and Infectious; subgroups of I to capture different detectability and infectiousness are explained in methods section. R: Recovered. T: Treatment. Compartments with subscript D denote temporary protection by having drugs

The two patches are represented graphically as two intersecting circles (Fig. 1). Force of infection for patch i (λ_i_) was defined as Eq. 1 so that when the level of connectedness between the two patches (*C*) was 0, λ had a different, independent value for each patch, and when C was 1, λ was identical for both patches.

1 $$\lambda_i={\left(1-C\right)\beta}_i\left(\frac{I_i}{P_i}\right)+\frac C2\frac{\left(\beta_1+\beta_2\right)\left(I_1+I_2\right)}{\left(P_1+P_2\right)}$$

where ${\upbeta }$ is the contact rate between mosquito and human, *I* is the combination of I_A_, I_C_, and I_U_ compartments, *P* is the total population in the respective patches.

The details of the model structure and interventions are in Additional file 1.

### Simulations

The two-patch model was simulated for several scenarios where one parameter of interest was varied at a time. The outcome metric measured from each patch in each simulation was whether or not a malaria elimination threshold, defined as “less than 1 infection per 1000 population per year” [8], was achieved one-year after the completion of a three-month MDA campaign. Since there were two patches, four outcomes were possible: achieving malaria elimination (a) in none of the patches, (b) in patch 1 only, (c) in patch 2 only and (d) in both patches.

**Fig. 2:**
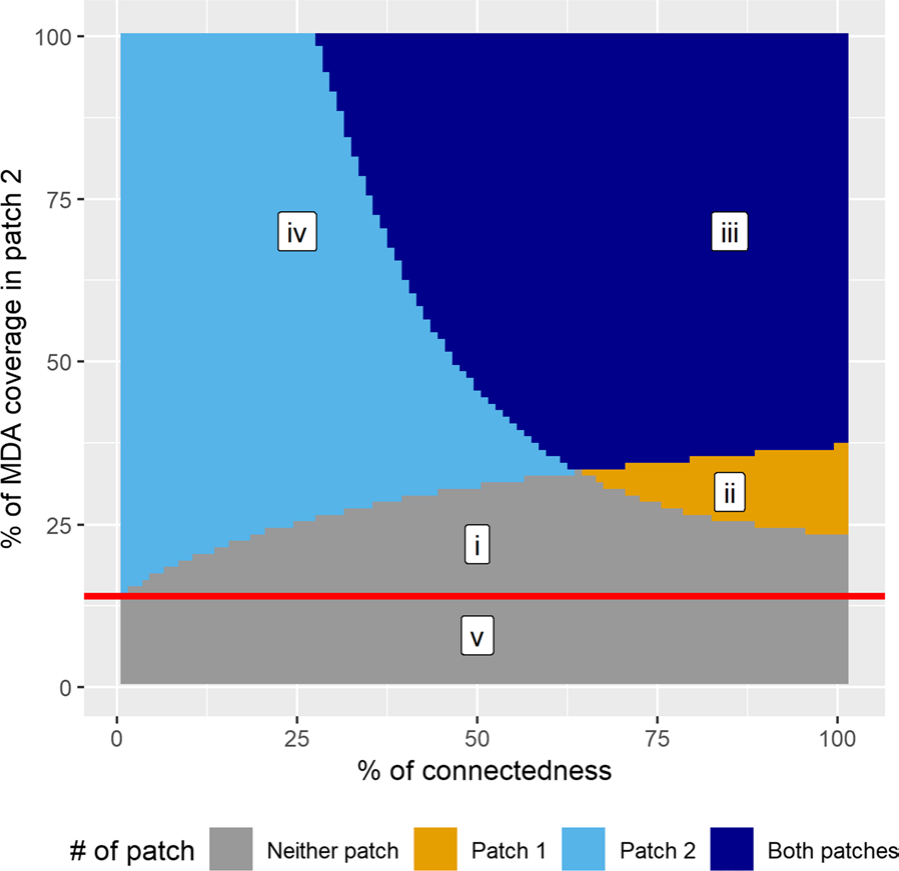
Illustrative example to guide in interpreting the surface plots in the result. On the left edge of the plot where connectedness is 0%, take the point where the grey or the orange colour changes to the light blue or the dark blue colour. A horizontal line from that point (red line in the figure) is the “baseline” or “minimal” MDA threshold for patch 2 to achieve elimination if it was isolated from other patches. Deviations from this line describe the assembly effect of patch 2. Lower-case roman numerals represent the different areas of the surface plot. The joint areas “i”+“ii” illustrate a negative assembly effect on patch 2, describing how an increase in MDA coverage would have to follow an increase in connectedness with another patch for elimination to be possible in patch 2. The joint areas “ii”+“iii” depict a positive assembly effect on patch 1, showing how an increase in connectedness with patch 2 can help eliminate transmission in patch 1 granted that it has a sufficiently high MDA coverage. This figure is merely an illustration and it is not an actual model result

The results were plotted on a two-dimensional surface plot. On the X-axis, the connectedness parameter, C, was increased from 0 to 100% with 1% incremental steps. The MDA coverage in patch 2 was increased from 0 to 100% on the Y-axis, while the MDA coverage in patch 1 is fixed at a particular value for each surface plot. These permutations resulted in over 10,000 simulations, the outcomes of which were summarized in the surface plots (e.g., Fig. 2).

These sets of simulations were repeated for the MDA coverage values in patch 1 from 0 to 90% with 10% increments and for the higher, identical, and lower pre-intervention disease intensities in patch 2.

### How to interpret the surface plots

Figure 2 serves as an example on how to interpret the figures in the result section. Different colours differentiate four possible outcomes: grey for not achieving malaria elimination in either of the patches (denoted by area “i” and “v” in the figure); orange for elimination in patch 1 only (area “ii”); light-blue for elimination in patch 2 only (area “iv”); and dark-blue for elimination in both patches (area “iii”). The required MDA coverage threshold for malaria elimination in patch 2 can be seen at the transition from the grey or orange area (area “i” or “ii”) to the light-blue or dark-blue area (area “iii” or “iv”). For a given malaria incidence in an isolated patch, there exists a specific “baseline” or “minimal” threshold of MDA coverage above which elimination could be achieved. The “baseline” MDA threshold for patch 2 in Fig. 2 is the MDA coverage that is required in patch 2 when the connectedness is 0%, indicated by the horizontal red line. Connectedness between the two patches is an indication of how much time humans from each patch spend in the other patch, with 100% connectedness indicating that the two patches are functionally the same patch and 0% connectedness indicating that there is no human movement between the patches (they are isolated). As connectedness increases (through human movement between the two patches), the MDA coverage threshold deviates from the red line. This is because the MDA coverage in patch 1 is high enough that there is a spill-over effect into patch 2 when the two patches are highly connected through human movement. The change in coverage threshold for successful intervention in a patch due to its connectedness to another patch is hereafter referred to as an “assembly effect”. The assembly effect can have either positive (i.e., protective) or negative implications for individuals in either patch. In Fig. 2, “i” + “ii” is the negative assembly effect for patch 2, where increasing connectedness with patch 1 increases the MDA coverage threshold required for elimination in patch 2. From the point of view of patch 1, “ii” + “iii” is the positive assembly effect – patch 1 does not achieve elimination when it is isolated, but it does after a certain level of connectedness.

## Results

**Fig. 3:**
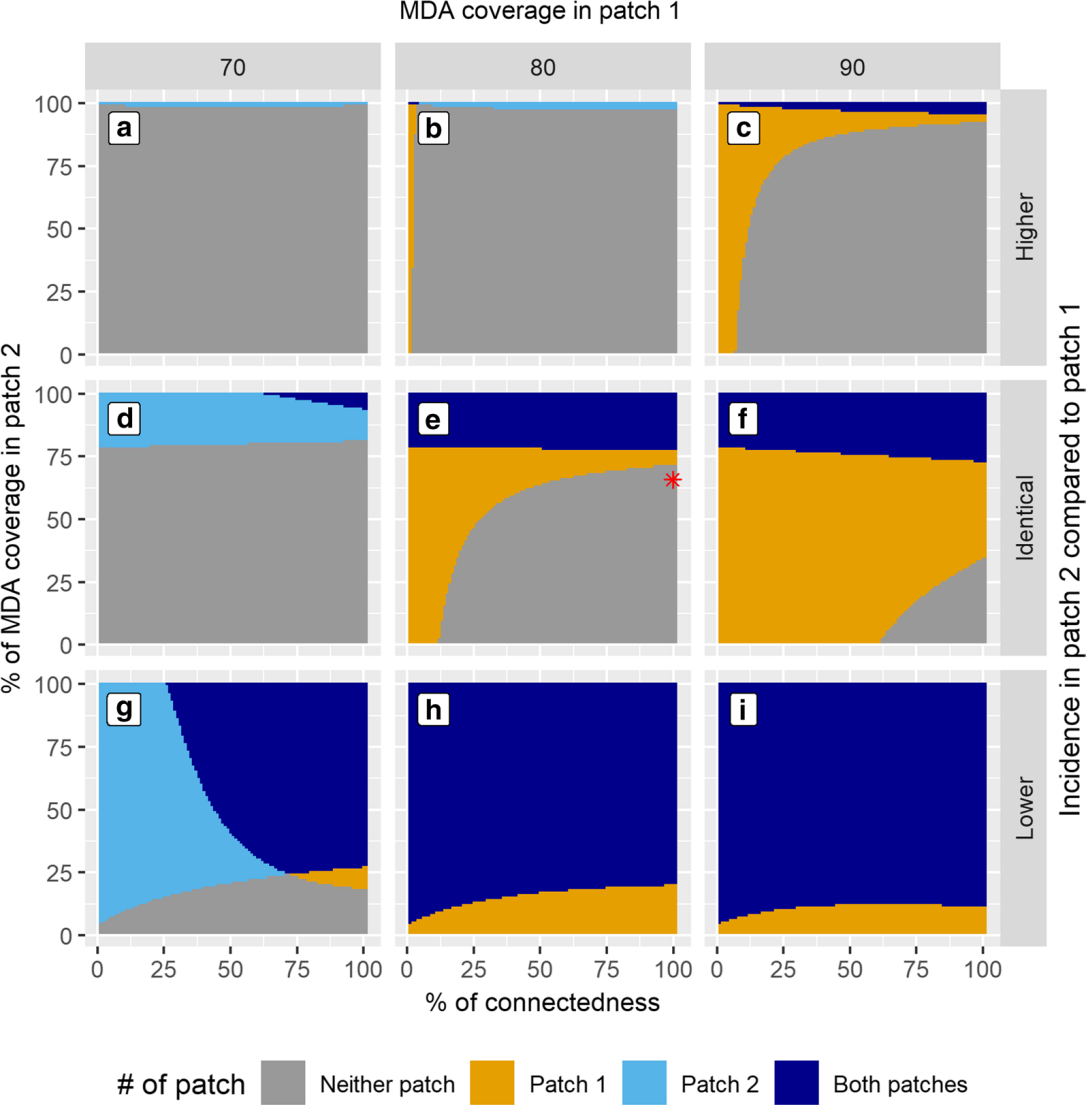
Achieving elimination in two connected patches by varying connectedness between the two populations (x-axis) and MDA coverage in the 2nd patch (y-axis) of each subplot. Columns represent different sets of MDA coverage in patch 1 (70%, 80%, and 90%, respectively). Each row represents the relative incidence level between the two patches. **a** MDA coverage in patch 1 is 70%, and patch 2 has higher pre-intervention incidence. No visually distinguishable assembly effect is found. **b** MDA coverage in patch 1 is 80%, and patch 2 has a higher pre-intervention incidence than patch (1) Patch 1 should achieve elimination on its own but did not achieve it because of its connectedness to patch 2 (negative assembly effect from the viewpoint of patch 1). **c** MDA coverage in patch 1 is 90%, and patch 2 has higher pre-intervention incidence. **d** MDA coverage in patch 1 is 70%, and both patches have identical pre-intervention incidence. Slight negative assembly effect from the viewpoint of patch (2) **e** MDA coverage in patch 1 is 80%, and both patches have identical pre-intervention incidence. Slight positive assembly effect from the viewpoint of patch 2. The red asterisk represents the combination of parameter values matching the MDA trial implementation described in Parker et al. Panel F: MDA coverage in patch 1 is 90%, and both patches have identical pre-intervention incidence. Increased positive assembly effect from the viewpoint of patch 2 compared to panel E. Panel G, H, I: MDA coverage in patch 1 is 70%, 80%, and 90% respectively. Patch 2 has lower pre-intervention incidence, and its baseline MDA coverage threshold is low. From the viewpoint of patch 2, there is always a negative assembly effect but its magnitude diminishes as the MDA coverage in patch 1 is increased from 70 to 90%

The simulation results in a collection of thirty plots (ten for each level of MDA coverage in patch 1, repeated for three relative pre-intervention disease intensities between the two patches). Only the last three MDA coverage levels (70%, 80%, and 90%) in patch 1 were focused on here, as the assembly effects in these scenarios are more pronounced for the demonstration purpose. The columns of sub-plots in Fig. 3 represent the MDA coverage in the patch 1 (column 1 = 70%, column 2 = 80%, column 3 = 90%); and the rows represent the relative pre-intervention disease intensities in patch 2 compared to patch 1 (top = higher, middle = identical, and bottom = lower).

### Assembly effect between two patches with the same incidence

In the middle row of Fig. 3, both patches have an identical pre-intervention incidence, requiring a baseline MDA threshold of 78% coverage to achieve elimination (when the patches are not connected). In Fig. 3d, there is a negative assembly effect for patch 2 (the grey area above the baseline MDA threshold) because of the increasing connectedness with patch 1, which has a relatively low MDA coverage (70%). However, the increasing connectedness is beneficial to patch 1 (a positive assembly effect). Despite patch 1 having 70% MDA coverage, and not being able to achieve elimination on its own, the increasing connectedness with patch 2 (when patch 2 has more than enough MDA coverage for itself e.g., 94% MDA coverage), makes elimination still attainable in patch 1 (dark blue triangle at the upper right corner).

An opposite effect is seen when patch 1 has higher MDA coverage (80 and 90%) than is necessary to achieve elimination on its own (Fig. 3e, f). In this scenario, patch 2 experiences a positive assembly effect, indicated by the extension of the dark blue areas below the baseline MDA coverage threshold of 78%. However, patch 1 experiences a negative assembly effect; as connectedness increases, elimination in patch 1 is not predicted to occur for low MDA coverage in patch 2 (grey area in the lower right corners) because less-than-optimal coverage in patch 2 prevents patch 1 from achieving elimination at those levels of connectedness.

When the pre-intervention transmission intensities are the same in the two patches, the resulting assembly effects are purely due to differences in intervention coverage. To quantify the total assembly effect in patch 2 in each plot, the area between the “baseline” MDA threshold line (the red line in Fig. 2) and the diverging MDA threshold for increasing levels of connectedness (i.e. area “i” + “ii” in Fig. 2) was integrated. The total effect is assigned positive if it is beneficial to patch 2, and it is assigned negative otherwise.

**Fig. 4:**
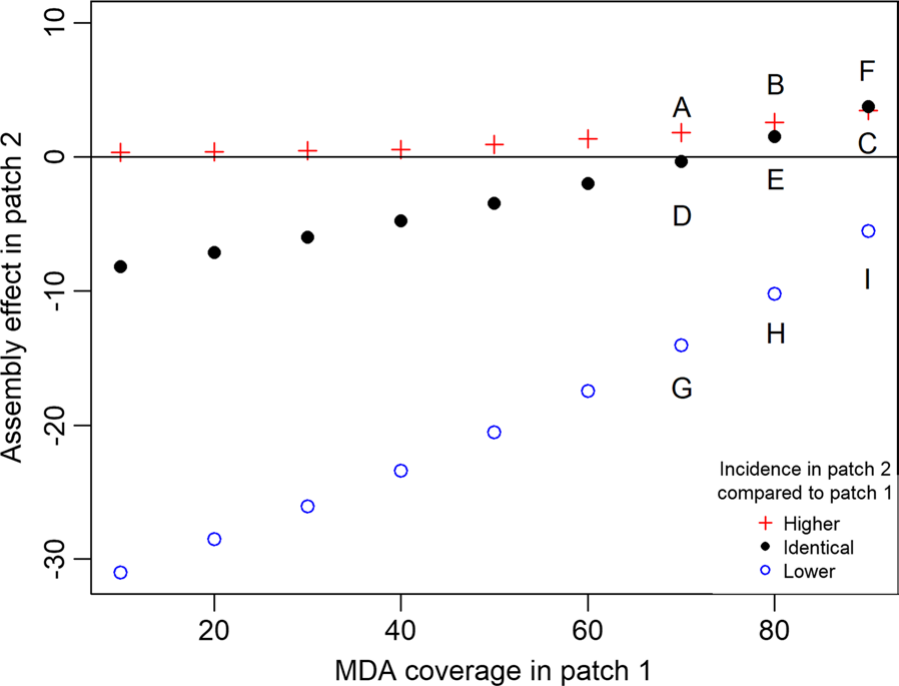
Total assembly effects in patch 2 where relative incidence is higher, identical, or lower compared to patch 1. The value of total assembly effects on the Y-axis was calculated by integrating the assembly effects in patch 2 over all levels of connectedness with patch 1. A, B, C, D, E, F, G, H, and I represent the assembly effects of respective panels in Fig. 3. Blue circles are analogous to the assembly effects in non-hotspot for different coverage in the hotspot. Red crosses represent the assembly effects when incidence in patch 2 is so high that MDA is not an effective intervention (i.e., Nearly 100% MDA coverage is required to achieve elimination in patch 2). Black dots represent the assembly effects when the two patches have identical incidence (i.e., assembly effects are the same from the point of view of both patches)

Figure 4 displays how the total assembly effect in a particular patch is modulated by its connectedness to the other patch for different relative incidence ratios. The total assembly effect in patch 2 increases with increasing intervention coverage in patch 1 (black dots in Fig. 4). The switch from negative to positive total assembly effect occurs at the “baseline” coverage threshold for the particular disease intensity shared by both patches (78% coverage in this case).

The model’s prediction was compared against results from an MDA trial described in Parker et al. [4] where a village failed to achieve elimination presumably due to a cluster of non-participation in the MDA. This scenario was modelled as a set of two contiguous patches with 100% connectedness and with identical incidence. One patch received approximately 80% MDA coverage and the other 64%, with the latter representing the non-participation cluster (details in Additional file 1). The model accurately predicted that neither patch would achieve elimination (the red asterisk in Fig. 3e).

### Assembly effect when two patches have different pre‐intervention incidences

#### Hotspot vs. non-hotspot

In the bottom row of Fig. 3, patch 2 has a 25% lower pre-intervention incidence compared to patch 1. This is analogous to a scenario where a low-incidence community (non-hotspot: patch 2) is connected to a high-incidence community (hotspot: patch 1). For this example, the following definition of malaria hotspots is used: “*geographical areas within a wider area of transmission in which the transmission intensity is significantly higher than the average level in the surrounding area of that setting and are widely observed in malaria-endemic regions*” [20]. When in isolation (no connectedness between patches), the MDA coverage threshold for elimination is very low at 5% for the non-hotspot, whereas it is 78% for the hotspot.

When MDA coverage in the hotspot is slightly below its required threshold for elimination (70% rather than the required 78%, Fig. 3g), both a negative assembly effect for the non-hotspot and a positive assembly effect for the hotspot are seen (areas similar to negative assembly effect for patch 2: “i” + “ii” and positive assembly effect for patch 1: “ii” + “iii” respectively in illustrative Fig. 2).

This suggests that when MDA coverage in the non-hotspot is high, and when the connectedness between hotspot and non-hotspot is high, elimination could be achieved in both patches despite the hotspot having less-than-optimal MDA coverage. For instance, when there is 60% connectedness, MDA coverage over 30% in the non-hotspot is predicted to result in elimination in both patches.

In panels H and I of Fig. 3, the hotspot has an adequate MDA coverage at 80 and 90% respectively. In those scenarios, the hotspot is predicted to always achieve elimination, regardless of the level of connectedness and the value of MDA coverage in the non-hotspot.

As seen in Fig. 4, non-hotspots (blue circles) will always experience a negative total assembly effect. However, the magnitude of the negative total assembly effect decreases with increasing coverage in the connected hotspot. The opposite is true for the positive total assembly effect gained by the hotspot (i.e., it increases with increasing coverage in the connected non-hotspot as seen in Additional file 1: Fig. S4). These trends suggest that the difference in transmission intensity is the main determinant of what types (positive or negative) of assembly effects can be observed.

In Fig. 3i, the required intervention threshold for the non-hotspot plateaus between 40 and 80% of connectedness. Further increase in the connectedness decreases the required intervention threshold slightly.

### Assembly effect when intervention is ineffective for the connected patch

An intervention may not be appropriate if the disease intensity is too high e.g., MDA may not work in a high-transmission setting unless a very high MDA coverage is achieved. This scenario was simulated in the first row of Fig. 3 by setting patch 2 as a high-transmission setting. In isolation, patch 2 would require almost 100% of MDA coverage, while patch 1 would require more than 78% coverage of MDA for elimination to be attainable. As a consequence of being connected to patch 2, the prospects for elimination in patch 1 would be greatly diminished (large negative assembly effect for patch 1 represented by grey areas in Fig. 3b, c).

## Discussion

In a single patch system, the success of an intervention depends on the pre-intervention disease intensity and the coverage of the intervention, provided the intervention is efficacious and its coverage is maintained for an adequate period. In the two-patch connected system, whilst those metrics are still relevant, the level of connectedness between the two patches (through human movement or travel) is a key determinant of the intervention’s success. The results illustrate how connectedness can bring an advantageous effect to one patch, while potentially being disadvantageous to the other. This effect is designated as the *assembly effect* and it is defined as:

The difference in the minimum intervention coverage required for a successful intervention in a specific patch when it is isolated versus when it is connected in some degree to another patch with potentially different disease intensity and/or different intervention coverage.

An assembly effect can be seen when connectedness is as low as 1%. Its magnitude and direction of effect depend on transmission intensity and intervention coverage in the adjacent area.

When connected patches have identical pre-intervention disease intensity, but different intervention coverage, the required threshold for successful intervention in each patch will equilibrate with increasing connectedness. In other words, the required intervention threshold in each connected patch approaches some average threshold values between them as their connectedness level is increased. A negative assembly effect (increment in the required threshold) occurs in a patch when it is connected to another patch that does not have enough intervention coverage to control its transmission intensity. At the same time, a positive assembly effect (decrement in the required threshold) may occur in the latter patch depending on how connected they are. Therefore, if one patch achieves a higher-than-optimal coverage of intervention, and its connected patch has a less-than-optimal coverage, it is still possible to attain a successful outcome in both patches, provided they are connected enough. This has implications for public health interventions in locations with low adherence. In settings where multiple communities or populations are highly connected, as long as a certain number of the populations achieve higher-than-optimal coverage, the remaining populations can have less-than-optimal coverage.

As countries move towards disease elimination and as disease transmission intensity distributions over space become extremely patchy [8], it becomes increasingly important to target disease hotspots with adequate intervention coverage. The results suggest that to achieve elimination, adjacent non-hotspot areas should not be left without interventions. Having some intervention coverage in the adjacent non-hotspots is also helpful when the optimal intervention coverage could not be achieved in the hotspots [21]. For highly connected patches, hotspots with sub-optimal intervention coverage are predicted to have a significant positive assembly effect because of the connectedness to the non-hotspot patches that have modestly increased MDA coverage above its required threshold (Fig. 3g and Additional file 1: Fig. S4).

Public health interventions that reduce transmission and target populations that are not in complete isolation will likely also result in an assembly effect. By considering the following: connectedness between populations, overall disease intensity, and adherence to the public health interventions being used, communicable diseases can more effectively be controlled and eliminated.

### Implications for the focal malaria interventions

The WHO has recommended MDA as a potential tool to accelerate malaria elimination but recommended its deployment only when core malaria interventions are already delivered in high-quality coverage and the area where it is implemented is in a very low transmission setting [8]. This study’s result aligns with the WHO’s recommendation by showing how it could be ineffective when applied before very low transmission is achieved. Once the very low transmission is achieved in many connected patches through improvement and maintenance of core malaria interventions, some patches with relatively higher incidence (hotspots) and relatively lower incidence (non-hotspots) could persist. In such a scenario, it would be tempting to target malaria hotspots with MDA. The results from this study suggest that targeting only malaria hotspots may not be enough. It is often challenging and resource-intensive to achieve high coverage for MDA [22, 23], and the imported asymptomatic infections from the connected non-hotspots could refuel transmission [24]. Therefore, when targeting hotspots in these scenarios, reinforcement of interventions in adjacent non-hotspots would benefit the hotspots because of the positive assembly effect and improve the chance of a successful elimination campaign. An example guideline for malaria elimination is described in Table 1.

**Table 1:** Example guidelines for a malaria elimination scenario

Background scenario	Suppose we are planning to eliminate malaria from a province with very low malaria transmission
Adequate core malaria interventions and identification of malaria hotspots	First, we must ensure the quality coverage of core malaria interventions such as early diagnosis and treatment, and long-lasting insecticide-treated nets (LLINs) in all villages within the province. We then need to identify the hotspot villages based on prevalence surveys or incidence reports
Information on connectedness	Depending on the budget and the available timeframe, connectedness between villages can be inferred in several ways. Remote sensing and GIS analysis may be used to infer connectedness through metrics such as distance, estimated population size, and estimated travel time. Human mobility surveys may be conducted to inform connectedness. GPS logger studies may be more expensive and labour intensive but could produce more detailed measures of connectedness. A multi-patch or individual-based model may be used to fit historical data of a similar area to yield an estimate of the connectedness
Optimisation of intervention coverage across hotspots and non-hotspots	Armed with some information on the connectedness between villages and the location of hotspots, we can strategize to ensure the efficiency and effectiveness of the focal MDA is optimized. All malaria hotspots should aim to reach an MDA coverage over the minimal threshold (i.e., 80% in most contexts). Non-hotspot villages that are connected to the hotspots should get an MDA coverage of at least 30%
Example calculation of MDA rounds required for the intended effective coverage	The MDA coverage which we have used here is the percentage of the target population who receives at least one round of MDA. Different total coverage levels could represent a different number of monthly rounds of MDA. In our model, the final MDA coverage of x% after 3 rounds means 1-(1-x)^(1/3)^ coverage in round 1. Therefore, if we achieve 70% of total MDA coverage after 3 rounds, we could say that 1 round of MDA will cover 33% of the total population. MDA coverage from our model can thus be operationalized into the number of MDA rounds. Using this information in our example scenario would mean that we could target malaria hotspots with three rounds of MDA while the non-hotspots which are connected to the hotspots could be provided with only one round of MDA

### Limitations

This model was developed as a theoretical framework to define the concept of the assembly effect in a general sense. There were many assumptions in the model structure and parameter values used. The way MDA was modelled in the compartmental system may not be an accurate representation of a real-world MDA. The model has so far been validated on a single scenario. Further rigorous validation and fitting would be required to use it as a predictive tool. The time point for measuring the outcome was arbitrarily set as one year after the completion of MDA. Results will vary depending on where this time point is set.

## Conclusions

Assembly effect is a meta-population version of the herd effect and it occurs between connected populations of potentially different disease intensities and/or intervention coverages. The ultimate impact of an intervention in an area depends on how well it is connected with neighbouring areas. Information on the level of connectedness between populations will inform efficient control and elimination strategies. For malaria, improving and maintaining core malaria interventions is the first step towards achieving very low transmission, which could be followed by an acceleration to elimination. In implementing accelerating activities such as MDA, targeting malaria hotspots alone may not be optimal. Having positive assembly effects on the hotspots by additionally implementing MDA with lower coverage on their connected non-hotspots will lower the required MDA coverage threshold in the hotspots and thus increase the feasibility of malaria elimination.

## Supplementary Information

**Additional file 1.** The model structure, the model validation, and the parameter values.

## Data Availability

The reproducible source code of the model can be found on https://github.com/SaiTheinThanTun/MDA_eff_pub.

## Abbreviations

IRS: Indoor residual spraying
ITN: Insecticide-treated nets
LLIN: Long lasting insecticide-treated mosquito nets
MDA: Mass drug administration
WHO: World Health Organization
