# Supplementary material for: The assembly effect: the connectedness between populations is a double‐edged sword for public health interventions
Source: Malar J. 2021 Apr 17;20:189. doi: 10.1186/s12936-021-03726-x (PMC8052750; doi:10.1186/s12936-021-03726-x)
Supplement: Supplementary file 1 — Additional file 1. The model structure, the model validation, and the parameter values. [file 12936_2021_3726_MOESM1_ESM.pdf]

Supplementary materials for “The assembly effect: the connectedness between populations is a double-edged sword for public health interventions”

Table of Contents

List of Authors 2

Methods 2

Model structure 2

Model validation 3

Figures 5

Tables 8

Table 1: Parameter values and descriptions 9

References 10

List of Authors

Sai Thein Than Tun, Daniel M. Parker, Ricardo Aguas, Lisa J. White

Methods

Model structure

The general model structure for a patch can be seen in Figure 1 which is mathematically represented by the set of equations below.

$$\frac{dS_i}{dt} = \mu P_i - \mu_{out} S_i + \omega R_i - \lambda_i S_i + \omega_D S_{Di} - m_i S_i$$

$$\frac{dI_{C_i}}{dt} = \mu_C P_i - \mu_{out} I_{C_i} + p_S(1-\tau)\lambda_i S_i + p_R(1-\tau)\lambda_i R_i + p_R(1-\tau)\lambda_i I_{U_i} + p_R(1-\tau)\lambda_i I_{A_i} - \nu_C I_{C_i} - m_i I_{C_i}$$

$$\frac{dI_{A_i}}{dt} = \mu_A P_i - \mu_{out} I_{A_i} + (1-p_S)\lambda_i S_i + (1-p_R)\lambda_i R_i + (1-p_R)\lambda_i I_{U_i} - p_R \lambda_i I_{A_i} + \nu_C I_{C_i} - \nu_A I_{A_i} + f \nu_T T_i - m_i I_{A_i}$$

$$\frac{dI_{U_i}}{dt} = \mu_U P_i - \mu_{out} I_{U_i} - \lambda_i I_{U_i} - \nu_U I_{U_i} + \nu_A I_{A_i} - m_i I_{U_i}$$

$$\frac{dR_i}{dt} = -\mu_{out} R_i - \omega R_i - \lambda_i R_i + \nu_U I_{U_i} + \omega_D R_{D_i} - m_i R_i$$

$$\frac{dT_i}{dt} = -\mu_{out} T_i + p_S \tau \lambda_i S_i + p_R \tau \lambda_i R_i + p_R \tau \lambda_i I_{U_i} + p_R \tau \lambda_i I_{A_i} - \nu_T T_i + m_i\left(I_{C_i} + I_{A_i} + I_{U_i}\right)$$

$$\frac{dS_{D_i}}{dt} = -\mu_{out} S_{D_i} + \omega R_{D_i} - \omega_D S_{D_i} + m_i S_i$$

$$\frac{dR_{D_i}}{dt} = -\mu_{out} R_{D_i} - \omega R_{D_i} + (1-f)\nu_T T_i - \omega_D R_{D_i} + m_i(R_i)$$

where $P_i = S_i + S_{D_i} + I_{C_i} + I_{A_i} + I_{U_i} + R_i + R_{D_i} + T_i$.

The description and assumed values of the parameters can be found in Table 1.

The two patches are connected through the λ term (see Figure 1 in the main manuscript) which is influenced by the level of connectedness, C, as described by the equation below.

$$\lambda_i = (1 - C)\, \beta_i \left(\frac{I_i}{P_i}\right) + \frac{C}{2} \frac{(\beta_1 + \beta_2)\,(I_1 + I_2)}{(P_1 + P_2)}$$

Where $C \in (0,1)$.

Therefore, when C=0,

$$\lambda_1 = \beta_1 \left(\frac{I_1}{P_1}\right), \lambda_2 = \beta_2 \left(\frac{I_2}{P_2}\right)$$

And when C=1,

$$\lambda_1 = \lambda_2 = \frac{0.5(\beta_1 + \beta_2)(I_1 + I_2)}{P_1 + P2}$$

For brevity, all the sub-compartments of *I* are not shown in the equation. β is the contact rate between mosquito and human, adjusted by the effectiveness and coverage parameters of insecticide treated nets ($\zeta_{ITN}, \kappa_{ITN}$). Coverage and effect of early diagnosis and treatment is modelled through the parameter τ. Mass drug administration (MDA) is modelled for three months during which $m_i = -\frac{\ln(1-\kappa_{m_i})}{3}$. m_i moves individuals from S and R to their respective compartments with active drug, and individuals from I to T (Figure 2).

Model validation

The parameters for the model are calibrated based on the data from Parker et al.[2] In order to do so, we simulated a scenario with the MDA coverages corresponding to the two parts of the village as in [2]. Human biting rate and the size of the patches were assumed to be the same.

In the result of this scenario analysis (Figure 3), the red line indicates that no elimination threshold would be achieved in the part of the village with lower MDA coverage (64%), at all level of connectedness. But because the two patches were from a single village, the connectedness would be close to 100%. At this connectedness

level, both parts of the village will not achieve the elimination threshold. Therefore, the model can explain why there were clinical cases throughout the village 12 months after the completion of the MDA. Table 1 lists of all other model parameter values.

Figures

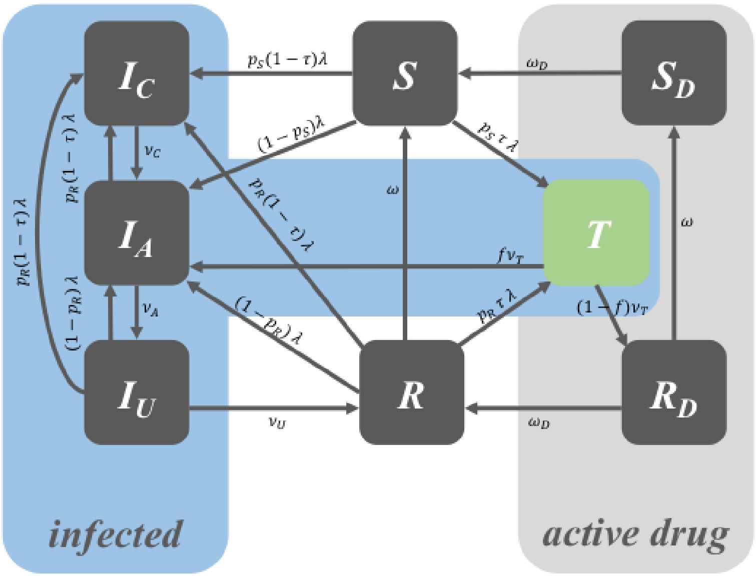

Figure 1: General structure of the compartmental model. Reproduced from our previous manuscript Tun et al. 2017

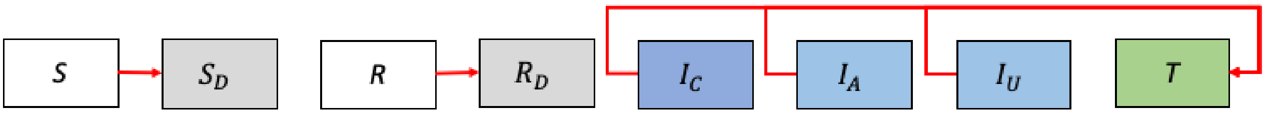

Figure 2: Consequence of MDA, revised from Tun et al 2017

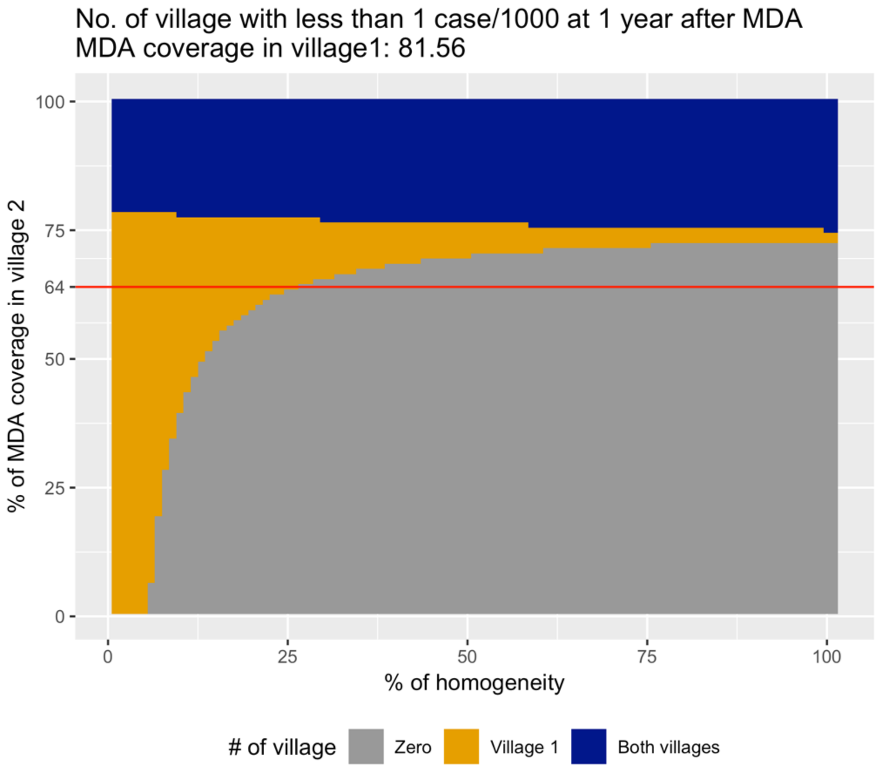

Figure 3: Result of the model calibration based on Parker et al. 2019

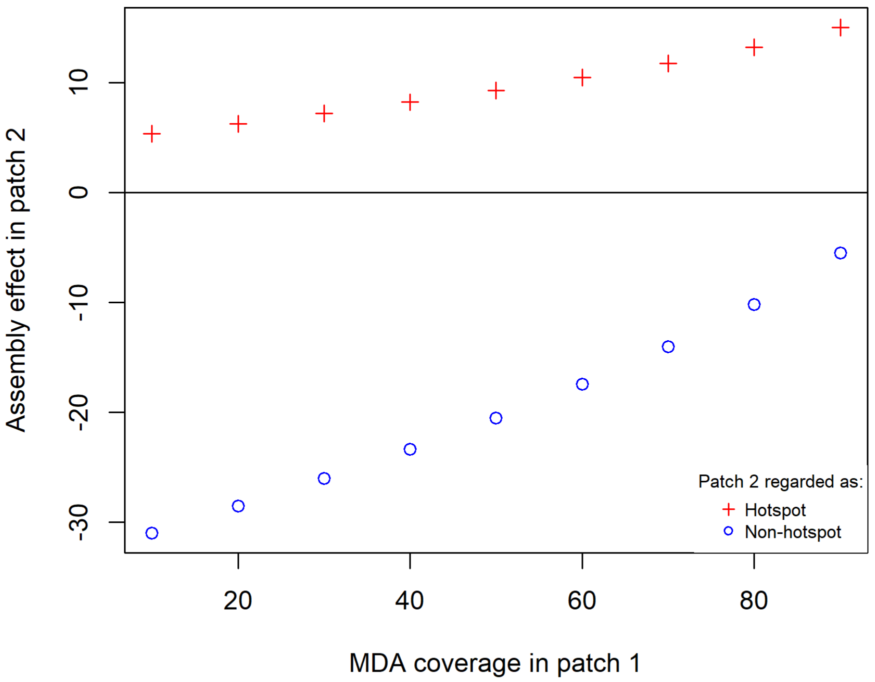

Figure 4: Assembly effects between a hotspot and a non-hotspot. The non-hotspot has 25% lower pre-intervention incidence compared to the hotspot.

Tables

#	Name	Description	Value	Unit	Reference
1	ζ_{ITN}	Effectiveness of ITN (insecticide treated nets), proportion of new infections averted due to ownership of ITN	0.30	proportion	[3]
2	κ_{ITN}	Coverage of ITN	0.70	proportion	-
3	κ_{MSAT}	Coverage of MSAT to prevent case importation from other areas	0.90	proportion	-
4	μ	Birth/death rate	1/69	/year	[4]
5	μ_A	Rate of importation of asymptomatic patent cases from other areas	1	/year/1000	-
6	μ_C	Rate of importation of clinical cases from other areas	1	/year/1000	-
7	μ_{out}	Death rate + emigration rates for malaria cases	-	-	-
8	μ_U	Rate of importation of asymptomatic non-patent cases from other areas	1	/year/1000	-
9	ν_A	Rate of transition from asymptomatic patent state (IA) to asymptomatic non-patent state (IU)	365/60	/year	[5]
10	ν_C	Rate of relief from clinical symptoms in absence of treatment	365/3	/year	[6]
11	ν_U	Rate of transition from asymptomatic non-patent state (IU) to recovered state (R)	365/100	/year	[7]
12	ν_T	Recovery rate after treatment with anti-malarial drug	365/14	/year	[8]
13	ξ_A	Sensitivity of the detecting an asymptomatic, patent (microscopically detectable) case with MSAT	0.87	proportion	-
14	ξ_C	Sensitivity of the detecting a Clinical case with MSAT	0.99	proportion	-
15	ξ_U	Sensitivity of the detecting an asymptomatic, non-patent (microscopically undetectable) case with MSAT	0.44	proportion	[9]

16	ρ_A	Relative infectivity of super-microscopic asymptomatic infections compared with clinical infections	0.55	proportion	[10]
17	ρ_U	Relative infectivity of sub-microscopic asymptomatic infections compared with clinical infections	0.17	proportion	[10]
18	ω	Rate of immunity loss	½	/year	-
19	ω_D	Rate of loss of protection by anti-malarial drug	365/30	/year	-
20	p_R	Proportion of all immune new infections that are clinical	0.20	proportion	[11]
21	p_S	Proportion of all non-immune new infections that are clinical	0.90	proportion	[5]
22	f	Proportion of failed treatment	0.05-0.30	proportion	-

Table 1: Parameter values and descriptions

References

1 Tun STT, von Seidlein L, Pongvongsa T, *et al.* Towards malaria elimination in Savannakhet, Lao PDR: mathematical modelling driven strategy design. *Malaria Journal* 2017; **16**: 483.

2 Parker DM, Tun STT, White LJ, *et al.* Potential herd protection against Plasmodium falciparum infections conferred by mass antimalarial drug administrations. *eLife* 2019; **8**: e41023.

3 Sochantha T, Hewitt S, Nguon C, *et al.* Insecticide-treated bednets for the prevention of Plasmodium falciparum malaria in Cambodia: a cluster-randomized trial. *Trop Med Int Health* 2006; **11**: 1166–77.

4 WHO. Life expectancy and Healthy life expectancy: Data by WHO region. http://apps.who.int/gho/data/view.main.SDG2016LEXREGv?lang=en (accessed Oct 29, 2019).

5 Collins WE, Jeffery GM. A retrospective examination of sporozoite- and trophozoite-induced infections with Plasmodium falciparum: development of parasitologic and clinical immunity during primary infection. *Am J Trop Med Hyg* 1999; **61**: 4–19.

6 Church LW, Le TP, Bryan JP, *et al.* Clinical manifestations of Plasmodium falciparum malaria experimentally induced by mosquito challenge. *J Infect Dis* 1997; **175**: 915–20.

7 Eyles DE, Young MD. The duration of untreated or inadequately treated Plasmodium falciparum infections in the human host. *J Natl Malar Soc* 1951; **10**: 327–36.

8 Adjuik M, Babiker A, Garner P, *et al.* Artesunate combinations for treatment of malaria: meta-analysis. *Lancet* 2004; **363**: 9–17.

9 Das S, Jang IK, Barney B, *et al.* Performance of a High-Sensitivity Rapid Diagnostic Test for Plasmodium falciparum Malaria in Asymptomatic Individuals from Uganda and Myanmar and Naive Human Challenge Infections. *Am J Trop Med Hyg* 2017; published online Aug 7. DOI:10.4269/ajtmh.17-0245.

10 Slater HC, Ross A, Ouédraogo AL, *et al.* Assessing the impact of next-generation rapid diagnostic tests on Plasmodium falciparum malaria elimination strategies. *Nature* 2015; **528**: S94-101.

11 Collins WE, Jeffery GM. A retrospective examination of secondary sporozoite- and trophozoite-induced infections with Plasmodium falciparum: development of parasitologic and clinical immunity following secondary infection. *Am J Trop Med Hyg* 1999; **61**: 20–35.
